# Early-Life Metabolic Traits and Physical Fitness in Tarahumara, Mennonite, and Mestizo Adolescents from Northern Mexico

**DOI:** 10.3390/nu15143208

**Published:** 2023-07-19

**Authors:** Raúl J. Nájera-Longoria, Irene Leal-Berumen, Yunuen S. Rangel-Ledezma, Angel Licón-Trillo, Verónica Moreno-Brito, Everardo González-Rodríguez, Miguel Conchas-Ramírez, Imelda G. Alcalá-Sánchez

**Affiliations:** 1 Faculty of Physical Culture Sciences, Autonomous University of Chihuahua, Chihuahua 31125, Mexico; jnajera@uach.mx (R.J.N.-L.); yrangel@uach.mx (Y.S.R.-L.); 2 Faculty of Medicine and Biomedical Sciences, Autonomous University of Chihuahua, Chihuahua 31125, Mexico; ileal@uach.mx (I.L.-B.); alicon@uach.mx (A.L.-T.); vmoreno@uach.mx (V.M.-B.); evegonzal@uach.mx (E.G.-R.); 3 Faculty of Law, Autonomous University of Chihuahua, Chihuahua 31160, Mexico

**Keywords:** Tarahumara, Mennonite, Mexican Mestizo, health risks, physical fitness, metabolic syndrome, adolescent

## Abstract

The WHO identifies high BMI, high blood pressure, and high fasting plasma glucose as chronic disease risk factors, whereas physical fitness is identified as a protective behavioral factor. This study responds to the rising interest in assessing metabolic factors and physical activity within young populations of Mestizo, Tarahumara, and Mennonite from Chihuahua Mexico, due to its strong relationship with disease development and low well-being. A cross-sectional study was conducted with 201 teenagers from rural towns in Northern Mexico, and relationships between physical fitness and cardio-metabolic risk related to anthropometric, glycolipid, and vascular function factors were assessed. ANOVA-tested differences among ethnic groups using physical fitness as a grouping variable and measures of cardio-metabolic risks were used as dependent variables. A stepwise regression analysis allowed us to identify the best predictors for physical fitness. Clinical risk factors were analyzed by ethnic group and sex. No differences were found among ethnic groups in physical fitness and cardio-metabolic health risks; sex differentiated higher health risks related to behavioral factors, since young women showed lower physical fitness across ethnicities. Clinically, the Mestizo sample showed higher numbers of individuals with one risk factor. Mennonites showed a high frequency of anthropometric and fitness health risks with low glycolipid and vascular risks. Tarahumara had fewer risk factors as compared with both Mestizo and Mennonite. Rural populations are harder to reach, both for health assessment and intervention; health professionals must work close to local community organizations to gain access.

## 1. Introduction

According to the Centers for Disease Control and Prevention (CDC), in the USA, obesity has increased almost three times in adolescents (12–19 years) in the last 30 years [1]. The 2018 National Survey of Health and Nutrition of Mexico reports that 36.8% of adolescents are either overweight or obese. Chihuahua state in Northern Mexico has 9.3% of the total country’s population with this phenotype [2], being acknowledged as a population health problem [3]. Overweight and obesity can be defined as a caloric imbalance, which means expending fewer calories compared to the ones consumed [4]. Childhood obesity is defined as a body mass index (BMI, kg/m^2^) greater than or equal to the 95th percentile according to specific sex-age-related growth charts [5]. Factors contributing to overweight include genetic susceptibility, environment, and behavior, the latter having the advantage of being modified by physical activity and healthy nutrition [6]. Obesity in young people has immediate and long-term effects, such as low self-esteem, sedentary behavior, and risk of having pre-diabetes, increased blood pressure, and hyperlipidemia. In the long term, those conditions eventually lead to metabolic syndrome [7], diabetes mellitus, cardiovascular diseases, and dyslipidemia disorders [8].

Over the past decades, there has been a rising interest in assessing the eating habits and physical activity within young populations of Tarahumara, Mennonite, and Mestizo from Chihuahua Mexico [8,9,10], due to its strong relationship with disease development and low well-being. Modernization among Tarahumara and Mennonite communities can increase the intake of processed food and may lead to losing physical fitness due to physical inactivity in younger populations; both changes can contribute to seriously growing health problems such as diabetes and cardiovascular disease in the future. Previous reports about the Mexican state of Chihuahua’s population, indicate that 70% of the sample were overweight or obese in Tarahumara [11] and according to the criteria of the National Cholesterol Education Program, over 18% of the sample shows metabolic syndrome in the Mennonite group [12].

Diet quality and physical inactivity can drive obesity or malnutrition, among other health problems in children and adolescents. Increased processed food intake and regular aerobic physical activity decreases may significantly impact the well-being of Tarahumara, Mennonite, and Mestizo’s younger populations. Increases in physical fitness through regular aerobic physical activity relate to better health and reduced mortality [13,14].

Physical fitness results from regular aerobic physical activity. A set of measurable attributes allowing performing physical activity includes measures of body composition, cardiorespiratory endurance, muscular fitness, and musculoskeletal flexibility; those attributes are indicators defining physical fitness [15,16].

Physical fitness seems to protect against early vascular changes and cardiovascular risk in obese young children [13]. This study aimed to assess differences in anthropometric and biochemistry indicators of health among Tarahumara, Mennonite, and Mestizo, youths as a function of their physical fitness.

## 2. Materials and Methods

### 2.1. Subjects and Study Design

A cross-sectional study was performed in rural high schools, from Chihuahua State, Mexico (cities of Guachochi, Cuauhtémoc, and Carichí).

To increase participation and inclusiveness for ethnicity and sex in all studied communities, we publicly recruited participants gaining access through local authorities. Regular channels of communication in the community were used with written, oral, and personal invitations to participate in both Spanish and Raramuri languages. Local authorities helped to gain access to participation. We offered participation incentives such as individual health reports for every participant; we also offered group reports whenever authorities and community representatives were interested.

All school authorities and parents received information about the study and all requirements for children’s voluntary participation. Written consent forms were provided to parents and tutors.

Inclusion criteria for all measures were having written consent from parents, understanding all instructions, fasting for at least 8 h, and voluntary acceptance for each test.

The convenience sample we used reflects those who were available in each community providing signed consent forms. All children with parental authorization also accepted every assessment voluntarily after being informed about each procedure.

Data were processed from 12-to-19-year-old students with complete information for ethnicity, sex, and physical fitness variables. The ethnicity for each participant was identified by social workers in school records.

The resulting sample was 201 individuals (selected by inclusion criteria from a previous main study of 1155 participants) distributed as shown in Table 1. We excluded individuals with missing data for each analysis.

### 2.2. Anthropometric Measures and Body Composition

Barefoot, standing height was measured with a manual stadiometer (Seca, Model 206). Participants wore light clothing and were barefoot when body mass was determined using a calibrated digital scale (Tanita, Model TBF 300, Tokyo, Japan). BMI was calculated. BMI CDC percentile was used for classification [1].

### 2.3. Blood Sample

Peripheral blood was collected in the supine position by venipuncture from the antecubital fossa by trained persons. Blood (4 mL) was collected into EDTA tubes and placed on ice, while another 6 mL was collected into SST tubes and allowed to clot for 20–30 min.

After fasting for blood sample collection and 30–60 min before running the physical fitness tests, scholars had a small breakfast consisting of liquid yogurt, one apple, and a small piece of sweet wheat bread.

### 2.4. Glucose and Lipids

Glucose sampling was performed in triplicate using the glucose GOD-PAP method (YSI 2300 Stat Plus, Yellow Springs Instrument, Yellow Springs, OH, USA). The average of the 3 glucose measurements was used in all analyses, and according to the WHO, normal values for the young population were established as 3.9 to 5.5 mmol/L.

Lipid profile, including measures of total cholesterol (CHOD-PAP method) (normal values less than 4.4 mmol/L), high-density lipoprotein cholesterol (HDL-C, normal values greater than 1.04 mmol/L), low-density lipoprotein cholesterol (LDL-C = CT-(TG/5)-HDL-C or CT-VLDL-HDL-C), and triglycerides (TGs) were determined through enzymatic assays (Human^®^, Mannheim, Germany) using an automatized biochemical analyzer (Prestige 24i ^®^, Tokyo Boeki^®^ Medical System LTD, Japan) (normal values less than 1.9 mmol/L). The atherogenic index was calculated through the formula Atherogenic Index = total cholesterol/HDL-C (normal values for males > 4.0 and females > 3.5 [17,18], which is used as a predictive cardiovascular risk factor [18]. The same cut-off values for both males and females were used since sex differences among children and adolescents have been observed as non-significant [19].

### 2.5. Physical Fitness

Flexibility: The sit and reach test allowed us to determine the flexibility of the lower back and hamstring muscles. Sitting on the floor, legs stretched out and barefoot placed against the measurement box. The subject reaches as far as possible forward with palms facing downwards and arms extended [20,21].

Strength: Arm, leg, and back strength measurements were carried out with a dynamometer with 660 pounds capacity (Baseline Dynamometer model 12-0403, Wayne, NJ, USA), testing postures and movements were followed as described by Ten Hoor, Musch [22]. The back, arm, and leg measurements were repeated three times and the mean ± standard deviation (SD) was reported. Values were then converted to kg.

Aerobic capacity: The multistage 20-m shuttle run test allowed the estimation of the VO_2_ max, with scores having a moderate-to-high criterion-related validity for estimating maximum oxygen uptake (r p = 0.66–0.84); this test is considered a useful field alternative to lab tests for estimating cardiorespiratory fitness [23,24] and it has been and is currently still in use to estimate aerobic fitness [25].

The test required running 20 m back and forward according to a rhythm stipulated by a prerecorded beep sound. Then, the following equation was used to determine the individual aerobic capacity: VO_2_ max (mL O_2_ min^−1^ kg^−1^) = 5.85 × Velocity − 19.45, where velocity is the maximal velocity reached at the end of the test.

### 2.6. Statistical Analysis

We present all results stratified by ethnicity and sex. Mean ± SD was used in normally distributed data, while the median was used for not normally distributed data.

We conducted a series of analyses of one-way variance (ANOVA) examining ethnicity and physical fitness as independent variables, and a Tukey test to assess statistical significance. Body composition, heart functions, and metabolic/biochemical measures were used as dependent variables to test differences between ethnic groups.

Comparisons were tested using Chi^2^ test values to assess significant differences among means of physical fitness measures by ethnicity, and in percentages on incidence of abnormal values for measured variables among ethnic samples. Simple linear stepwise regression was used to identify the best-predicting equation; as an exploratory technique, it was considered useful to eliminate variables superfluous for physical fitness to tighten future research. Pearson correlations were included for metabolic and physical fitness variables. SPSS v 21.0 Armonk, NY, USA) was used for all statistical analyses.

## 3. Results

### 3.1. Characterization of the Total Population

The studied teenagers (n = 201) included 61.69% males (n = 124), with an overall mean ± SD age of 14.48 ± 1.570 for males, and 13.97 ± 1.376 for females (Table 2).

Three ethnic groups were identified as sharing different social and cultural characteristics, backgrounds, or experiences, including language and behaviors; 56.2% (n = 113) were Mestizo, 24.9% (n = 50) were Tarahumara, and 18.9% (n = 38) were Mennonite.

Significant differences were observed between sex in height for Mestizo males (mean 1.62 m) vs. females (mean 1.53 m), [t (110,1) = 4.984, *p* = 0.000], and for Tarahumara height, with males being 1.59 m vs. females 1.53 m, [t (48,1) = 2.846, *p* = 0.007]; overall, males had higher height than females.

### 3.2. Physical Fitness

To analyze differences between males and females, and differences among ethnicity by physical fitness, a compound variable was calculated with individuals having complete data on the following measures: strength (arm, leg, and back), flexibility, and VO_2_ max. Table 3 shows descriptive statistics for those measures, by sex and ethnicity.

Using data from flexibility, arm strength, leg strength, back strength, and VO_2_ max, a new variable “physical fitness” was construed. Descriptive statistics for physical fitness are included by ethnicity and sex in Table 4.

A Chi^2^ test showed no differences among ethnic groups on physical fitness measures (Chi^2^ (1, 368) = 372.653; *p* = 0.423). However, in a physical fitness by sex analysis, males showed significantly higher physical fitness than females across ethnicity (Chi^2^ (1; 184) = 248.824; *p* = 0.001).

**Figure 1:**
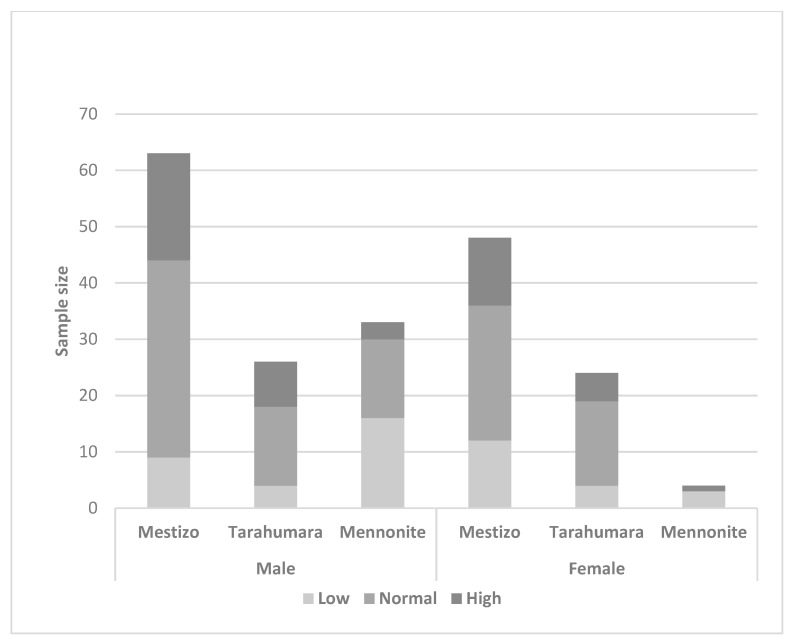
Classification of physical fitness by ethnicity and sex.

Based on the physical fitness score, boundaries were defined for low, normal, and high fitness using 50 and 25± percentiles. Percentages of the sample by fitness condition are shown in Figure 1.

### 3.3. Analysis of Variance

A series of one-way ANOVA was performed to compare the effect of ethnicity on biochemistry measures.

A one-way ANOVA revealed that there were statistically significant differences in systolic blood pressure [F (1195) = 16.618, *p* = 0.000]; glucose [F (1195) = 5.743 = 16.618, *p* = 0.017]; TG [F (1, 195) = 4.339, *p* = 0.039], and cholesterol [F (1195) = 4671, *p* = 0.032] between at least two groups.

Tukey’s HSD test for multiple comparisons found that the weighted mean value for SBP was significantly different between Mennonite [SBP mean 120.45(13.460)] and Mestizo [SBP mean 108.98(12.116)], (*p* = 0.000, 95% C.I. = 5.62, 16.27], and between Mennonite and Tarahumara [SBP mean 107.98(10.253)], (*p* = 0.000, 95% C.I. = 6.21, 18.37). There was no statistically significant difference in SBP between Mestizo and Tarahumara (*p* = 0.784, 95% C.I. = −3.43, 6.12).

Tukey’s HSD test for multiple comparisons found that the weighted mean value of glucose was significantly different between Mennonite [G, mean 77.500(7.6149)] and Mestizo [G, mean 82.823(8.186)], (*p* = 0.004, 95% C.I. = −8.880, −1.408), and between Mennonite and Tarahumara [G, Mean 84.980(9.0045)], (*p* = 0.000, 95% C.I. = −11.680, −3.145). There was no statistically significant difference in glucose between Mestizo and Tarahumara (*p* = 0.249, 95% C.I. = −5.620, 1.084).

Tukey’s HSD test for multiple comparisons found that the unweighted mean value of TG was significantly different between Mennonite [TGl, mean 80.1211(34.9336)], and Tarahumara [TGl, Mean 104.3520(41.9606)], (*p* = 0.006, 95% C.I. = −43.3034, −5.9087). There was no statistically significant difference in triglyceride between Mestizo [TGl, mean 94.0071(34.0544)] and Tarahumara (*p* = 0.233, 95% C.I. = −24.8550, 4.5150), and between Mestizo and Mennonite (*p* = 0.096, 95% C.I. = −1.9312, 30.8033).

Tukey’s HSD test for multiple comparisons found that there was no statistically significant difference in the mean value of cholesterol between Mestizo [Chol., mean 148.15(26.685)] and Tarahumara [Chol., mean 141.20(27.463)], [(*p* = 0.298, 95% C.I. = −4.14, 18.28)]; Mestizo and Mennonite [Chol., mean 139.13(31.821)], [(*p* = 0.123, 95% C.I. = −2.09, 22.90)], as well as between Tarahumara and Mennonite (*p* = 0.846, 95% C.I. = −10.94, 17.61).

The homogeneity assumption of variances among Mestizo, Tarahumara, and Mennonite was met for systolic blood pressure, minimum cardiac frequency, glucose, cholesterol, TG, HDL-C, LDL-C, VLDL, and Atherogenic Index. However, homogeneity of variance assumption was not observed for DBP (Lavene statistical *p* = 0.004) or for waist–hip index (*p* = 0.045).

### 3.4. Distribution of Cases with Normal and Abnormal Variables by Ethnicity and by Sex

Assessment of the OMS three global top risk variables of metabolic risks for years of life lost in 2040 (high blood pressure, high BMI, and high fasting plasma glucose) showed no significant ethnicity differences in our sample.

**Figure 2:**
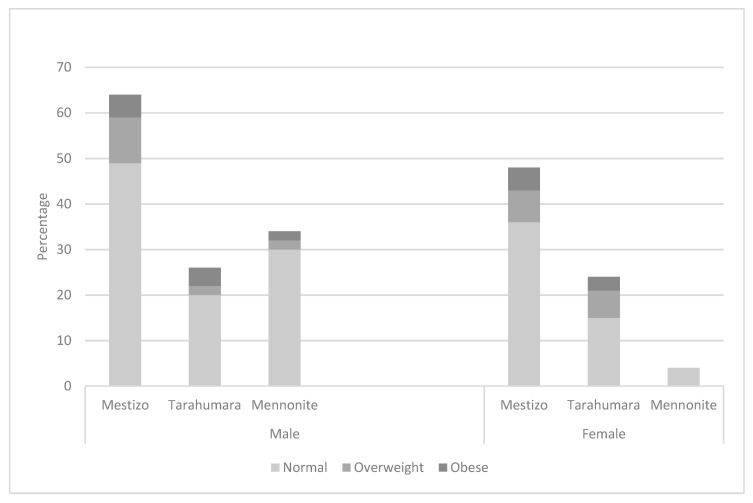
Body mass index by ethnicity and sex.

No differences in obesity and overweight BMI were observed among ethnic groups for males (Chi^2^ (1, 4) = 4.325; *p* = 0.364) and females (Chi^2^ (1, 4) = 3.039; *p* = 0.551). However, 10 males were overweight (11.3%) and 5 obese (8.9%), whereas 13 (16.9%) females were overweight and 8 (10.4%) were obese (Figure 2).

**Figure 3:**
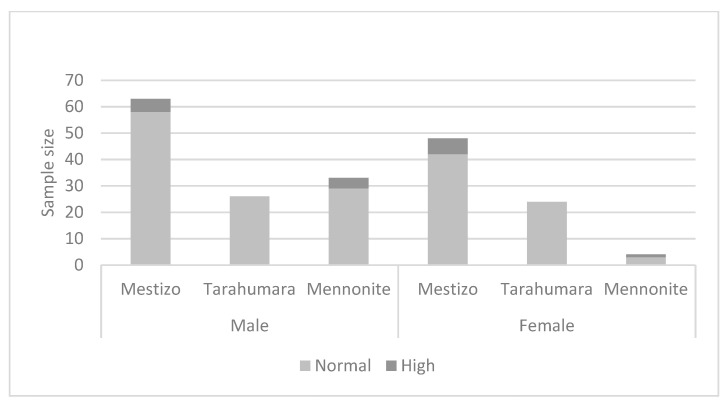
Systolic blood pressure by ethnicity and sex.

SBP showed elevated values for 9 males, as well as for 7 females, with non-significant differences among ethnic groups (males Chi^2^ (1, 2) = 3.187; *p* = 0.203; females Chi^2^ (1, 2) = 4.248; *p* = 0.120), (Figure 3).

**Figure 4:**
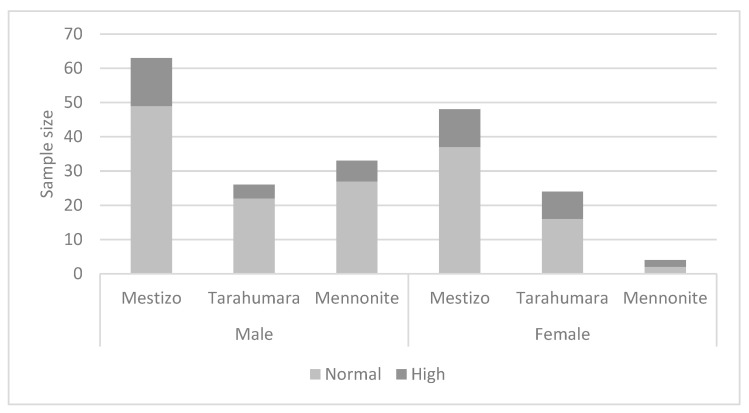
Diastolic blood pressure by ethnicity and sex.

DBP also showed no significant differences among ethnic groups, 24 males and 21 females showed elevated values (males Chi^2^ (1, 2) = 0.608; *p* = 0.738); females Chi^2^ (1, 2) = 1.925; *p* = 0.382), (Figure 4).

**Figure 5:**
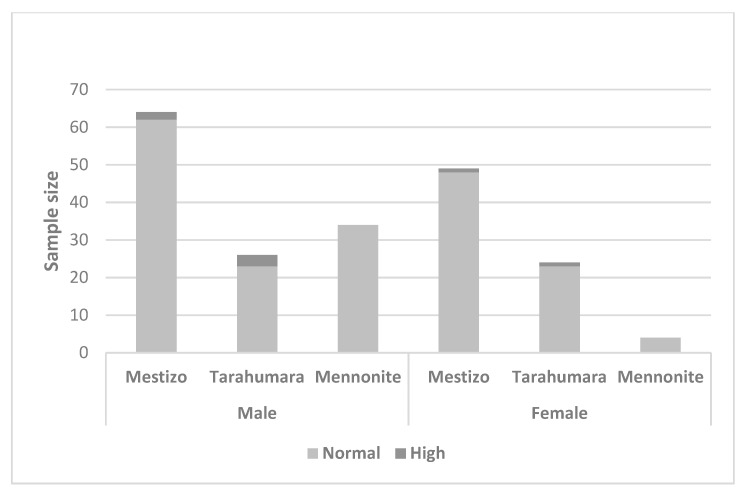
Glucose by ethnicity and sex.

Finally, no significant differences in glucose were observed among ethnic groups, 5 males (4%) and 2 females (2.6%) showed increased glucose values (males (Chi^2^ (1, 2) = 5.350; *p* = 0.069); females (Chi^2^ (1, 2) = 0.400; *p* = 0.819), (Figure 5).

Frequencies of individual risk factors were calculated using all variables assessed in this study. We calculated the total number of variables showing abnormal values for each participant to identify clinical variables of cardio-metabolic risk related to anthropometric, glycolipid, and vascular function factors. Table 5 shows percentages of individuals by ethnicity and sex, having from one, up to four, abnormal values on measured variables in this study.

The male Mestizo group showed the highest percentage (19.35%) of individuals having one, up to three, cardio-metabolic risk variables; fewer Tarahumara individuals (6.44%) showed elevated values on risk variables, as compared to Mennonite (8.86%). Male Mestizos also showed a higher percentage of individuals (35.47%) having abnormal values on additional risk variables, as compared with Tarahumara (14.43%) and Mennonite (21.5%), with Tarahumara having a lower percentage of affected individuals.

Mestizo females affected with one, up to four, risk factors were also on the highest percentage (28.55%), followed by Tarahumara (15.57%), and Mennonite (3.89%) with the lowest percentage of affected females; Mestizo females with additional risk factors, from one, up to four, showed also the highest percentages of affected individuals (49.33%), followed by Tarahumara (15.57%), and Mennonite with the lowest percentage of affected females (3.89%). Individuals affected with additional risk factors were observed in Mestizo females (49.33%), followed by Tarahumara (25.96%), and Mennonite (3.89%) being the least affected ones (Table 5).

### 3.5. Regression Analysis

We used stepwise regression to build a model, not to test one. According to this purpose, the stepwise criteria were probability of F to enter ≤ 0.050, probability of F to remove ≥ 0.100. However, since for this analysis, only cases with complete data were selected, our sample size for linear regression was small (n = 21) (Table 6). Therefore, a two halves analysis was not conducted, limiting our conclusions.

The overall regression was statistically significant (R^2^ = [0.219], F (1,19) = [5.341], *p* = [0.032]), with LDL-C as the best predictor of physical fitness (β = −0.468, *p* = 0.032).

All other variables (Height in meters, BMI, SBP, DBP, glucose, cholesterol, TG, HDL-C, LDL-C, VLDL, and Atherogenic Index) did not significantly predict physical fitness and were excluded from the exploratory model, as shown in Table 7.

A Pearson correlation coefficient was computed to assess the linear relationship between variables included in the regression analysis.

There were negative correlations between physical fitness and LDL-C (r = −0.468, *p* = 0.016); glucose and SBP (r = −0.458, *p* = 0.019); cholesterol and height in meters (r = −0.442, *p* = 0.022); HDL-C and height in meters (−0.477, *p* = 0.014); HDL-C and SBP (r = −0.439, *p* = 0.023); and between Atherogenic Index and HDL-C (r = −0.512, *p* = 0.009).

Positive correlations were observed between DBP and BMI (r = 0.405, *p* = 0.034); DBP and SBP (r = 0.778, *p* = 0.000); HDL-C and cholesterol (r = 0.538, *p* = 0.006); LDL-C and cholesterol (r = 0.897, *p* = 0.000); Atherogenic Index and cholesterol (r = 0.436, *p* = 0.024); and between Atherogenic Index and LDL-C (r = 0.619, *p* = 0.001) (Table 8).

## 4. Discussion and Conclusions

The Sustainable Development Goals defined by the general assembly of the United Nations set Target 3.4 as “By 2030, reduce by one-third premature mortality from non-communicable diseases through prevention and treatment and promote mental health and well-being” [26]. Three main risk factors associated with premature death were identified in the global population: high blood pressure, high BMI, and high fasting plasma glucose. Moreover, the protective effects of physical fitness have been demonstrated against those health risk factors [27].

The existence of multiple metabolic and behavioral risk factors among the Mexican population demands appropriate prevention strategies to halt the progress of the non-communicable disease burden within the region.

To improve health in Northern Mexico rural populations, identifying health risk factors is a necessary task. The purpose of this study was to evaluate relationships between physical fitness and health measures, comparing three ethnic samples of youth living in Northern Mexico.

Results showed no significant differences among ethnic groups for physical fitness and cardio-metabolic health risks but sex-differentiated higher health risks related to behavioral factors since young women showed lower physical fitness across ethnicities than young men.

From the exploratory model, using measures to assess metabolic health, LDL was the most significant predictor of physical fitness; however, due to the small sample, this result is not conclusive. We need to test this result with a sample having more participants from both sexes.

We observed significant differences between Mennonite and the other two ethnic groups, showing higher values in SBP [120.45(13.460)] and lower values in fasting glucose [77.500(7.6149)] when compared with Mestizo’s SBP [108.98(12.116)], as well as Mestizo’s glucose [82.823(8.186)]. Mennonite also has higher values as compared with Tarahumara’s SBP [107.98(10.253)] and glucose [84.980(9.0045)]. In the same trend, Tarahumara’s TG [104.3520(41.9606)] was significantly higher than Mennonite’s [80.1211(34.9336)] but was non-significantly different from Mestizo [94.0071(34.0544)].

Those findings might be related to ethnic differences, where Mennonites stand apart from Mestizo and Tarahumara.

Cholesterol was non-significantly different between Mestizo [148.15(26.685)], Tarahumara [141.20(27.463)], and Mennonite [139.13(31.821)], all mean values were below standard normal values for children and teenagers (below 170 mg/dL).

Higher percentages of afflicted individuals, with multiple health risks among Mestizos (35.4%), as compared with Mennonites (21.5%) might be related to a combination of lower physical fitness and different and unhealthier feeding practices related to ethnicity. According to this, Tarahumara had a lower percentage of affected individuals (14.43%).

Mestizo females affected with one, up to four, risk factors were also on the highest percentage (28.55%), followed by Tarahumara (15.57%), and Mennonite (3.89%) with the lowest percentage of affected females; Mestizo females with additional risk factors, from one, up to four, showed also the highest percentages of affected individuals (49.33%), followed by Tarahumara (15.57%), and Mennonite with the lowest percentage of affected females (3.89%). Individuals affected with additional risk factors were observed in Mestizo females (49.33%), followed by Tarahumara (25.96%), and Mennonite (3.89%) being the least affected ones. However, those differences might be affected by the small number of female participants in Tarahumara and Mennonite samples.

Elevated cholesterol (39.30% n = 79 individuals) and TG (29.35% n = 59 individuals) were the two most prevalent risk factors. Mestizos showed higher numbers of individuals with one risk factor. Mennonites showed a high frequency of anthropometric and fitness health risks with low glycolipid and vascular risks. Tarahumara participants were the lowest in health risks as compared with both Mestizo and Mennonite individuals.

More than nineteen percent (19.35%) of male Mestizos showed metabolic risk factors, followed by Tarahumara males (6.44%). Less than nine percent (8.86%) of Mennonite individuals have one or more risk factors for chronic diseases.

Our group’s main study determined the prevalence of metabolic syndrome (MS) risk factors in 1155 scholars that included 59.7% Mestizos, 24.7% Tarahumara, and 15.7% Mennonite. The prevalence of MS in the sample was higher in Tarahumara adolescents (12.65%) compared to Mestizo (11.95%) and Mennonite (7.15%), according to the ATP III criterion [28,29]. The significant statistical differences in the prevalence of MS in adolescents from the different ethnic groups in Chihuahua may include diet quality, behavior, physical fitness, and genetic (inherited polymorphisms) differences.

In Mexico, the prevalence of dyslipidemia, arterial hypertension, obesity, and diabetes mellitus in the child and adolescent population has increased to a great extent during the last decade [30].

We found different results in terms of overweight and obesity in the total study population (27.6%) to those reported by Cárdenas-Villareal et. al. (2010) conducted with 254 adolescents from the city of Monterrey, Nuevo León, México that showed 21% for overweight and obesity. These data are lower than the reported prevalence of both entities in adolescents by the 2018 National Health and Nutrition Survey, which describes 22.5% overweight and 13.9% obesity in young Mexicans between 12 and 19 years of age [31].

Currently, there is no consensus on the possible impact on public health of the identification of cardiovascular risk components at an early age, both in our country and in our state, and the importance of phenotypic differences among ethnic groups. Therefore, we encourage researchers to continue multidisciplinary studies to improve knowledge and strategies for the well-being of our young populations.

## 5. Limitations

This study included only the regions of Cuauhtémoc, Guachochi, and Carichí. The results exposed here could lead to an incomplete understanding of the current health and physical fitness condition of the ethnic groups living in Chihuahua, Mexico. Nutrition and habits were not included in this study, which could give a more integrated view regarding metabolic traits and risk factors.

Since our Mennonite and Tarahumara samples were small, with smaller numbers of females, our data may not be representative of the population in Northern Mexico. Obtaining access to this rural population was a difficult task, as well as getting consent from females. Rural populations are harder to reach, both for health assessment and intervention; health professionals must work close to local community organizations to gain access. It will be interesting to continue this study in collaboration with the local government to design health strategies designed for each community. Including the adult population could give us a better understanding of the health problems that affect each ethnic group, especially those related to metabolic chronic diseases.

## Figures and Tables

**Table 1:** Participants by sex and ethnicity.

			Ethnicity			Total
			Mestizo	Tarahumara	Mennonite	
Sex	Male	N	64	26	34	124
		%	51.6	21.0	27.4	100.0
	Female	N	49	24	4	77
		%	63.6	31.2	5.2	100.0
Total		N	113	50	38	201
		%	56.2	24.9	18.9	100.0

**Table 2:** Descriptive, stratified by sex and ethnicity.

Ethnicity		Sex	n	Mean ± SD
Mestizo	Age	Male	64	13.91 ± 1.24
		Female	49	13.57 ± 1.17
	Weight	Male	64	52.81 ± 12.25
		Female	48	50.04 ± 10.18
	Height	Male	64	162.10 ± 9.64 *
		Female	48	153.17 ± 9.03 *
	BMI	Male	64	20.002 ± 3.80
		Female	48	21.44 ± 4.80
Tarahumara	Age	Male	26	13.77 ± 1.24
		Female	24	14.29 ± 1.20
	Weight	Male	26	52.03 ± 9.64
		Female	24	52.07 ± 8.13
	Height	Male	26	159.77 ± 8.51 *
		Female	24	153.73 ± 6.20 *
	BMI	Male	26	20.35 ± 3.42
		Female	24	22.06 ± 3.47
Mennonite	Age	Male	34	16.12 ± 1.30
		Female	4	16.50 ± 1.915
	Weight	Male	34	67.59 ± 13.60
		Female	4	59.15 ± 7.49
	Height	Male	34	179.68 ± 9.04
		Female	4	173.25 ± 10.63
	BMI	Male	34	20.87 ± 3.47
		Female	4	19.80 ± 2.87

* Significant differences between males and females *p* = 0.05.

**Table 3:** Descriptive statistics for measures of physical fitness, by sex and ethnicity.

Sex	Ethnicity		n	Mean ± SD
Male	Mestizo	Flexibility	64	−0.49 ± 8
		Arm strength	64	14.66 ± 8.63
		Leg strength	64	81.33 ± 43.39
		Back strength	64	71.52 ± 35.81
		VO_2_ max	63	47.1 ± 5.35
		n	63	
	Tarahumara	Flexibility	26	0.21 ± 7.16
		Arm strength	26	11.63 ± 9.24
		Leg strength	26	74.50 ± 37.63
		Back strength	26	64.92 ± 32.87
		VO_2_ max	26	48.78 ± 3.98
		n	26	
	Mennonite	Flexibility	34	0.13 ± 8.04
		Arm strength	34	9.10 ± 6.63
		Leg strength	34	21.78 ± 22.37
		Back strength	34	33.72 ± 23.77
		VO_2_ max	33	45.10 ± 5.59
		n	33	
Female	Mestizo	Flexibility	49	1.05 ± 6.14
		Arm strength	49	9.24 ± 6.59
		Leg strength	49	48.96 ± 26.10
		Back strength	49	43.54 ± 22.21
		VO_2_ max	49	42.05 ± 3.84
		n	49	
	Tarahumara	Flexibility	24	1.54 ± 6.14
		Arm strength	24	7.77 ± 5.15
		Leg strength	24	48.60 ± 22.60
		Back strength	24	46.52 ± 22.14
		VO_2_ max	24	40.05 ± 3.67
		n	24	
	Mennonite	Flexibility	4	−3.82 ± 7.75
		Arm strength	4	7.62 ± 4.01
		Leg strength	4	14 ± 14.74
		Back strength	4	22 ± 3.94
		VO_2_ max	4	38.74 ± 5.15
		n	4	

**Table 4:** Sample for physical fitness analyses.

Ethnicity	Male		Female	
	n	Mean ± SD	n	Mean ± SD
Mestizo	64	167.02 ± 84.60	49	102.80 ± 51.61
Tarahumara	26	151.30 ± 81.15	24	104.43 ± 47.55
Mennonite	34	64.74 ± 50.25	4	39.80 ± 25.70

**Table 5:** Percentage of individuals with cardio-metabolic risk by ethnicity and sex.

	WHO Risk Variables (BMI, SBP and DBP, and Fasting Plasma Glucose)				Additional Risk Variables *			
	Males (n = 124)							
Number of variables w/Risk Values	1	2	3	4	1	2	3	4
Mestizo	16 (12.9%)	7 (5.65%)	1 (0.8%)	0	22 (17.74%)	13 (10.48%)	4 (3.22%)	5 (4.03%)
Tarahumara	4 (3.22%)	4 (3.22%)	0	0	10 (8.0%)	6 (4.83%)	1 (0.8%)	1 (0.8%)
Mennonite	8 (6.45%)	3 (2.41%)	0	0	14 (11.29%)	10 (8.0%)	2 (1.61%)	1 (0.8%)
	WHO Risk Variables				Additional Risk Variables *			
	Females (n = 77)							
Number of Variables w/Risk Values	1	2	3	4	1	2	3	4
Mestizo	16 (20.77%)	4 (5.19%)	2 (2.59%)	0	20 (25.97%)	6 (7.79%)	9 (11.68%)	3 (3.89%)
Tarahumara	7 (9.09%)	4 (5.19%)	1 (1.29%)	0	7 (9.09%)	5 (6.49%)	7 (9.09%)	1 (1.29%)
Mennonite	3 (3.89%)	0	0	0	0	3 (3.89%)	0	0

* Cholesterol, TG, HDL-C, LDL-C, VLDL, Atherogenic Index, flexibility, resistance, VO_2_ max, physical fitness.

**Table 6:** Linear regression descriptive statistics (n = 21).

Variable	Mean ± SD
Physical fitness	243.12 ± 129.84
Height in meters	1.57 ± 0.16
BMI	21.29 ± 6.23
SBP	108.5 ± 14.3
DBP	70.95 ± 8.18
Glucose	80.24 ± 7.05
Cholesterol	143.52 ± 23.53
TG	98.81 ± 37.21
HDL-C	45.52 ± 8.49
LDL-C	78.24 ± 19.83
VLDL	19.76 ± 7.44
Atherogenic Index	3.25 ± 0.52

**Table 7:** Excluded variables from ^a^ Physical Fitness Model.

Model	Beta In	T	Sig.	Partial Correlation	Collinearity Statistics
					Tolerance
Height in meters	0.053 ^b^	0.241	0.812	0.057	0.899
BMI	−0.100 ^b^	−0.474	0.641	−0.111	0.955
SBP	−0.145 ^b^	−0.698	0.494	−0.162	0.983
DBP	−0.195 ^b^	−0.959	0.350	−0.220	1.000
Glucose	0.015 ^b^	0.071	0.944	0.017	0.974
Cholesterol	0.454 ^b^	0.989	0.336	0.227	0.196
TG	0.200 ^b^	0.976	0.342	0.224	0.983
HDL-C	0.079 ^b^	0.369	0.717	0.087	0.931
VLDL	0.200 ^b^	0.976	0.342	0.224	0.983
Atherogenic Index	−0.037 ^b^	−0.142	0.889	−0.033	0.617

^a^ Dependent Variable: physical fitness, ^b^ predictors in the model: (Constant), LDL-C.

**Table 8:** Correlations among anthropometric, glycolipid, and vascular measures.

Variables	P Fitness	Height in m	BMI	SBP	DBP	Glucose	Cholesterol	TG	HDL-C	LDL-C	VLDL	At Index
P Fitness	1.000											
Height in meters	0.197	1.000										
BMI	−0.195	−0.543	1.000									
SBP	−0.080	0.338	0.332	1.000								
DBP	−0.189	0.160	* 0.405	* 0.778	1.000							
Glucose	−0.062	−0.283	−0.003	* −0.458	−0.323	1.000						
Cholesterol	−0.331	* −0.442	0.246	−0.333	−0.131	0.280	1.000					
TG	0.257	−0.006	0.263	−0.202	0.143	0.165	0.158	1.000				
HDL-C	−0.049	* −0.477	−0.043	* −0.439	−0.462	0.250	* 0.538	−0.139	1.000			
LDL-C	* −0.468	−0.319	0.212	−0.132	−0.012	0.163	* 0.897	−0.129	0.262	1.000		
VLDL	0.257	−0.006	0.263	−0.202	0.143	0.165	0.158	.	−0.139	−0.129	1.000	
At Index	−0.313	0.066	0.262	0.105	0.349	−0.002	* 0.436	0.314	* −0.512	* 0.619	0.314	1.000

* Significant correlation, probability 0.05.

## Data Availability

Not applicable due to the small sample.
